# Consumption and Informal Trade of Milk in the North of Antioquia (Colombia)

**DOI:** 10.1155/2024/6644328

**Published:** 2024-03-22

**Authors:** Richard Zapata-Salas, José F. Guarín, Leonardo A. Ríos-Osorio

**Affiliations:** ^1^School of Microbiology, University of Antioquia, Medellín, Antioquia, Colombia; ^2^Research Group in Health and Sustainability, Research Group in Veterinary Microbiology, University of Antioquia, Medellín, Antioquia, Colombia; ^3^Department of Agricultural Sciences, University of Antioquia, Medellín, Antioquia, Colombia; ^4^Research Group in Agricultural Sciences–GRICA (Acronym in Spanish), University of Antioquia, Medellín, Antioquia, Colombia

## Abstract

The excessive and irrational use of antibiotics to control bovine mastitis and the informal trade in milk with antibiotic residues are objects of great interest for public health, due to the problems associated with the consumption of milk with antibiotic residues on human, animal, and environmental health, and antibiotic resistance. The objective of this study was to understand the attitudes of dairy farmers towards the self-consumption of milk on the farm, the use of milk with antibiotic residues, and the formal and informal milk trade that generates risks for public health. Mixed methods: cross-sectional and grounded theory. Convergent triangulation design. The study was carried out in 9 dairy municipalities in the North of Antioquia, where 216 dairy farmers participated in the quantitative component; and 17 milk producers and 9 veterinarians took part in the qualitative component. A dairy farmer characterization survey was conducted, as well as a survey on the use and marketing of milk from cows with udder health problems and/or under antibiotic treatment. Semistructured interviews were conducted on the same subject. The variable “Intention to sell milk in the village when the dairy industry does not buy it due to high BTSCC” is associated with the BTSCC variable. The variable “Type of marketing reported” is associated with the CFU variable. 5 categories: self-consumption of milk, use of milk with antibiotics, informal milk trade, control of the dairy industry, and beliefs about the elimination of antibiotics in milk, were constructed to theorize about udder health and public health. Sociocultural, political, and economic factors affect decision making in dairy farmers regarding the use and marketing of milk from cows with mastitis and antibiotic residues. These attitudes and behaviors have public health implications.

## 1. Introduction

Milk and dairy products are foods consumed every day by people around the world [1]. According to the National Survey of the Nutritional Situation in Colombia (2010), 48.7% of Colombians consume milk [2].

In dairy production systems, mastitis is the most common bacterial disease [3]. Most antibiotics used in dairy cattle are directly related to interventions to restore udder health once an inflammatory process has been detected in the mammary gland [4, 5]. In the Netherlands, 22% of antibiotic use on dairy farms has been associated with the treatment of clinical mastitis and 44% with the treatment of dry cows [6]. Comparable proportions have been reported in Canada [7] and Ireland [8]. In Colombia, there are no studies on the use of antibiotics in dairy cattle.

The use of antimicrobials in animals is one of the promoters of bacterial resistance to antimicrobials [9, 10]. Therefore, the presence of antibiotic residues in milk is considered an emerging threat to global public health [11], animal health, and environmental health [12, 13]. The consumption of milk with antibiotic residues can generate toxic manifestations, even though these residues are found in low concentrations, due to their cumulative effect [2, 12]. Just bacterial resistance to antibiotics causes more than 700,000 deaths per year worldwide [13].

Milk may be contaminated with antibiotics due to a number of reasons including overuse of antibiotics, inability to identify treated animals, failure to apply antibiotics as prescribed, purchase of antibiotic-treated cows, and lack of knowledge or forgetfulness about the withdrawal period, which can lead to the trade of milk with antibiotic residues in the formal and informal trade. These reasons are dependent on human behavior, planning, and decision making on the farm [14].

Although in Colombia a formal milk channel to processing plants has been established, there is a significant volume of milk that goes to the informal trade, which, according to figures from the Ministry of Agriculture and Rural Development (MADR) reported in 2021, corresponds to 45% of the milk produced [15]. According to Guzmán, cited by MinSalud and FAO (2014), the reasons that favor this informal trade include consumer habits, a lower cost per liter of raw milk compared to pasteurized milk, remote areas where there are no collection centers, secondary and tertiary roads in poor condition that prevent the producer from reaching the collection centers, and the low interest of processors for small dairy farmers and their low production volumes [2].

The consumption of raw milk is common among the rural population and part of the general population. In the United States, between 35 and 60% of the population in dairy farming areas consumes raw milk [16]. Raw milk supporters base their consumption culture on beliefs, considering that raw milk is a product with a higher nutritional value than pasteurized milk [17]. In addition, they consider it to be a completely safe product [16]. Cultural rootedness is also influenced by the belief that it is a functional food in this state, where the consumer expects that unpasteurized milk can prevent and treat a wide spectrum of diseases, including heart disease, kidney disease, cancer, and lactose intolerance [16]. Feeding calves with antimicrobial-containing discarded milk is another common practice among dairy farmers in Europe and the Americas, with prevalence ranging from 33 to 87% [18]. This practice has been associated with increased antimicrobial resistance in the commensal microbiota of calves [19], impairment of the gastrointestinal tract and respiratory microbiome [20], and calf health [21].

The sanitary profile of raw milk in Colombia conducted in 2013 reported 5,078 raw milk marketers in the country, of which 246 marketers are in Antioquia, which corresponds to the absence of accounting in the self-consumption and trade of raw milk in 60.9% of the country's municipalities [2]. In terms of quality indicators, the national study found that only 0.7% of the milk analyzed met the criteria for total quality, the presence of pathogenic bacteria in raw milk, and 5.5% of the samples analyzed were positive for antibiotics, demonstrating the problems in the implementation of policies on udder health, quality in milk production, and public health [2].

In Colombia, there are no studies on udder health, self-consumption of milk, and informal milk trade in primary production from a sociocultural view of decision making by the primary dairy farmer and public health. The objective of this study was to understand the attitudes of dairy farmers towards self-consumption of milk on the farm, the use of milk with antibiotic residues, and the formal and informal milk trade that generates risks to public health.

## 2. Materials and Methods

### 2.1. Study Design

Mixed methods: cross-sectional and grounded theory. Convergent triangulation design [22].

### 2.2. Study Subjects

For the quantitative component, non-probabilistic sampling stratified by municipality was carried out, with a similar number of small, medium, and large dairy farmers. In total, 216 dairy farmers, located homogeneously across nine municipalities in the North of Antioquia, volunteered for the study. From each farm, the owner, manager, or head milker in charge of production was chosen who had complete knowledge of the management of the milk production, consumption, and marketing system.

Dairy farmers and veterinarians who attend the farms of the participating dairy farmers participated in the qualitative component. The number of participants was defined through a theoretical sampling by category saturation [22]. The saturation of preestablished and emerging categories was achieved with 17 dairy farmers. The selection of participants was carried out through maximum variation sampling to capture the maximum plurality of discourses typifying human reality in relation to our study: sex (males and females); age (young people from 20 to 26 years old, adults between 27 and 59 years old, and elderly from 60 years old); farm size (small, medium, and large); municipality (representatives of the 9 municipalities); educational level (no studies, elementary, high school, university student, technical and technological education, graduates, and specialist); and functions on the farm (owner, manager, milker, and trader). For the veterinarian population, saturation of preestablished and emerging categories was achieved with 9 veterinarians. Maximum variation sampling included sex (males and females); age (young people from 20 to 26 years old, adults between 27 and 59 years old, and elderly from 60 years old); farm size (small, medium, and large); municipality (representatives of the 9 municipalities); work experience (years); and employment relationship (employed at a milk collection/supply company or freelance worker).

### 2.3. Data Collection

An instrument of 10 questions was developed to characterize the educational level, production systems, and farm certification in good livestock practices, brucellosis, and tuberculosis. Additionally, an instrument with 4 questions on the use of milk with antibiotics and marketing and its association with biological indicators of udder health (BTSCC and CFU) was used. An initial face validity and content validity of the selected items were carried out with 4 experts in udder health, milk production, and public health to determine the relevance of the structure of the items, as well as their clarity, completeness to capture the required information, and accuracy of the variables in relation to the purpose of the study. These experts had doctoral training and/or more than 40 years of experience in the area, higher education and research, as well as publications in this field. In addition, there was a PhD researcher with experience in survey design and validation. Subsequently, 40 subjects from the study population evaluated the preliminary instrument to determine its comprehension, acceptability, and applicability.

A semistructured interview was developed based on the categories identified in two systematic reviews on udder health [23, 24]. The instrument underwent an initial appearance validation and a content validation of the selected themes. The interview inquires about the meanings, representations, and attitudes about self-consumption of milk, the use of milk with antibiotic residues, and the formal and informal milk trade that dairy farmers and veterinarians experience in their daily lives. The interview was conducted as follows: (a) contextualization of the study and informed consent, (b) self-consumption of milk, (c) use of antibiotics, and (d) formal and informal milk trade, in no particular order. Two to three face-to-face interviews were conducted with each participant according to the open, axial, and selective coding format of grounded theory [25].

### 2.4. Methodological Rigor Criteria of the Qualitative Component

The credibility, auditability, and transferability criteria were applied in the study [26].

The data on udder health indicators (BTSCC and CFU) were supplied by the dairy company to which the dairy farmer sells its milk, with the dairy farmer's prior approval. The laboratories are accredited under the NTC-ISO/IEC 17025: 2005 standard. For the raw database, BTSCC and CFU averages were calculated by taking biweekly data for each farm during the period from September 2019 to August 2020. This resulted in only one BTSCC and CFU per farm. The CFU and BTSCC variables are presented according to ranges based on Colombian regulations and adapted by Múnera-Bedoya et al. [27].

### 2.5. Analysis of the Information

Absolute and relative frequencies are described for the categorical variables. The BTSCC and the CFU were defined as dependent variables. The association between the survey variables and the dependent variables was evaluated using the Mann–Whitney U and Kruskal–Wallis H tests after verifying non-compliance with the assumption of normality, evaluated using the Kolmogorov–Smirnov test with Lilliefors correction. Data were analyzed using SPSS-IBM version 25® software. In all the analyses, a statistical significance of *p* ≤ 0.05 was taken.

All interviews were recorded. The recordings were transcribed with the Transcribe version 4.13.0 software, reviewed, and corrected manually to guarantee their total accuracy. Subsequently, they were imported into the ATLAS.ti software version 22. The interviews were analyzed following the stages of open, axial, and selective coding. Open coding allowed conceptualization based on the abstract representation of the phenomena described by the participants. In this sense, a code was assigned to each text fragment, which was compared according to their common characteristics and meanings. This coding stems from theoretical categories preestablished by the authors and from the words of the participants. Axial coding arises from the codes created in open coding. Here the categories and subcategories, and their relationship according to their properties and dimensions were established. With selective coding, the central category (Fate of milk with antibiotic residues) was determined, and all the categories were integrated to propose a theoretical construct. The central category was defined in accordance with the following criteria, proposed by Strauss and Corbin: (I) that all main categories are related to the central one; (II) that each category or most of them provide indicators to the concept; (III) that the relation between categories allows for a solid explanation; (IV) that it explains the contradictory or alternative cases to the central idea of the category; and (V) that the concept is refined when it integrates others. The theoretical outline allowed for the elimination of the excess data and the completion of the categories still underdeveloped through additional theoretical sampling. The theory formed was validated by comparing it with the raw data, and after the participants acknowledged the theoretical proposal as a close conceptualization of their realities [25].

**Figure 1:**
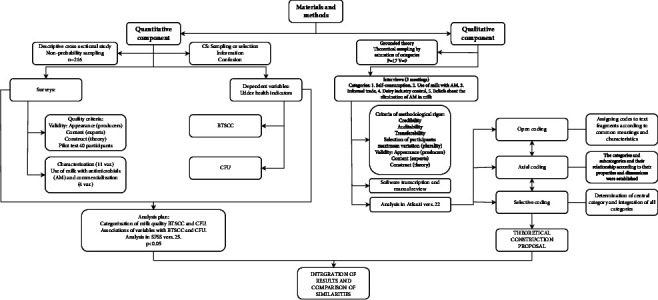
Materials and methods.

The analysis of the integrated results was conducted in accordance with the mixed methods proposal with convergence triangulation design, through the comparison of similarities and integration of the qualitative and quantitative results in a matrix and the compared discussion of both paradigms [22].  Figure 1 summarizes the materials and methods of the study (see Figure 1).

## 3. Results

Dairy farmers participating in the study are distributed among young people (5.1%), adults (77.8%), and older adults (17.1%). Dairy farmers participating in the study are distributed among young people (5.1%), adults (77.8%), older adults (17.1%), women (6%), and individuals with secondary (81.4%), technical-technological (7.4%), and professional-postgraduate training (11.4%). Most of the population is classified into socioeconomic strata 2 (49.5%) and 3 (27.8%). Half of them perform all functions in the production system and are associated with a cooperative. In most of the farms, the mechanical vacuum system in the paddock (39.8%), mechanical system in the parlor (28.2%), and manual (28.2%) are used as the milking system. Most of the dairy farmers' farms are leased (45.4%), owned (40.3%), family-owned (8.3%), and owned plus another leased part (6%). 83.3% of the farms are not certified in good livestock practices. 69% correspond to farms certified as free of brucellosis and 63.4% to farms certified as free of tuberculosis.

While only 10.2% of the farms produce milk of good or excellent sanitary quality (BTSCC), 67.6% have deficient sanitary quality. On the other hand, most of the farms, 54%, produce milk with excellent hygienic quality, and only 22% represent farms with deficient hygienic quality of milk (see Table 1).

An association was identified between those who intend to sell milk in the village when the dairy industry does not buy it by high BTSCC and BTSCC, being higher for those who intend to take their milk to the informal trade. The BTSCC and CFU are higher for those who sell their milk in informal trade (see Table 2).

### 3.1. Categories and Subcategories in the Analysis of Informal Milk Consumption and Trade That Have an Impact on Public Health

**Figure 2:**
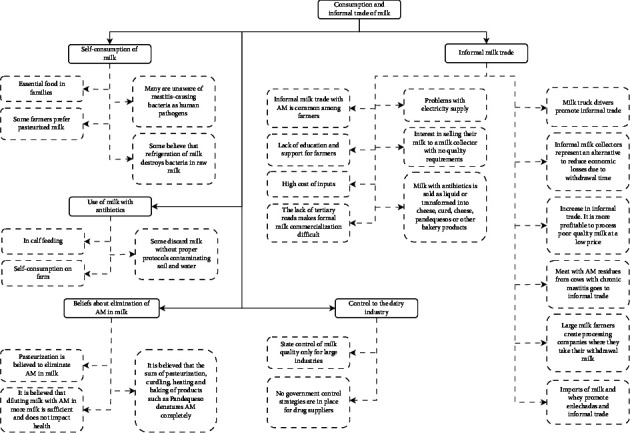
Category tree in the analysis of informal milk consumption and trade in the North of Antioquia.

The qualitative analysis of the interviews with dairy farmers (DF) and veterinarians (V) allowed us to recognize 5 categories: self-consumption of milk, use of milk with antibiotics, informal milk trade, control of the dairy industry, and beliefs about the elimination of antibiotics in milk (see Figure 2)

#### 3.1.1. Self-Consumption of Milk

Milk produced on the farm is an essential food for dairy families.

DF1. If you have cows and don't drink milk, then why do you have them? Milk cannot be missing in the kitchen, at home they consume milk for breakfast, lunch, and dinner.

DF2. Regarding culture, I have drunk a lot of milk, I have been raised in the country all my life, and milk has always been there, it has never been missing. We drink the milk we produce ourselves, of course, boiled!

Part of the dairy farmers prefer pasteurized milk considering the health risk from raw milk consumption.

DF12. We almost always consume pasteurized milk, from the farm, very little of it.

Some producers are aware of diseases that can be transmitted to humans through milk; however, they are less aware of mastitis-causing bacteria that could cause disease in humans.

DF1. There are diseases that cattle have that can be transmitted to a person, right? But I don't know what bacteria could be transmitted from mastitis.

Self-consumption of milk is a traditional practice. For some, it is reliable to drink milk from the farm itself. Some mistakenly consider that tank cooling kills bacteria in milk; therefore, it is consumed without heat treatment.

DF4. We consume milk from the farm, no more. I don't buy milk, even if it's processed and all, but it's not the same as the milk from the farm. And for the same reason, because we always had it in good terms, of very good quality, and even more with those cooling tanks, it keeps the milk cold, and kills bacteria.

#### 3.1.2. Use of Milk with Antibiotics

The withdrawal milk from cows treated with antibiotics, when it does not go to the informal trade, is used to feed calves, or it is processed into by-products for the family's self-consumption.

V2. The antibiotics that we can administer to the cow will be eliminated by the mammary gland. So the implication is something very serious in my opinion, because the dairy farmer, in order to avoid losing the milk that the cow is producing 10, 15, or 20 liters, curdles the milk for home consumption, or gives it to calves, or sells it to cheese factories.

Veterinarians do not agree with this practice, and they recommend discarding it to dairy farmers, but it seems to have become a tradition to feed calves with milk from cows undergoing antibiotic treatment.

V5. They use the milk of cows treated with antibiotics to raise the calves. Obviously, it is not recommended, but it is what is done the most, it is the most common and it happens everywhere.

DF3. They say that we cannot give milk with antibiotics to the calves, but we give it to them so as not to lose so much money, because sometimes it is a lot!

However, there are also dairy farmers who, due to their ethics, discard the milk from a cow that is being treated with antibiotics. This good attitude requires advice, since the discarding of milk with antibiotics must also be carried out with protocols that guarantee that this milk will not contaminate water and soil, since the environment is closely related to human and animal health.

DF1. The drug is so bad, and, in that milk, it is so strong that it is not even good for calves.

V4. There are many people who simply milk it and dump it. How? I don't know, I haven't asked them how they dispose of that milk because it is still a hazard, it can get into the water, into the ground. I think it is a serious matter because resistance is getting worse and worse, not only in cows, dogs, or cats, but also in us.

It is becoming more and more common in the rural population, the presentation of different non-specific health problems related to the consumption of milk with antibiotics, hormone, and drug residues.

V7. How many consultations at the human medical level are there for non-specific diarrhea? How many consultations at the medical level are there of situations in which the doctor is informed about the appearance of itching in an inexplicable way? Listen, even more serious, hormonal treatments; remember that hormonal treatments, most of them come out through the udder.

#### 3.1.3. Control of the Dairy Industry

Veterinarians of milk collectors and dairy farmers affirm that government control over milk quality is only applied to large dairies and processors. Small processors have no control whatsoever. This political behavior has interests in the indicators and a disinterest in the informal milk trade.

V6. We return to the same control issue; there is no control. Only large companies are controlled because, for a regulatory entity, it counts how many dairy farmers it can supervise. Is it clear? Then, if I take a company that is Jorgito's and has five thousand dairy farmers, and I go to the small one that has only 10, for the regulators, the large one is more useful to them due to the number of dairy farmers. For them it has more impact to say that we were supervising a company with five thousand or one thousand dairy farmers than this one with 10 or 100. Small companies and cheese companies have no control because the regulatory body is not interested in supervising a small company.

DF6. There are other companies and there are other cheese factories, I call them clandestine, when they work, I guess, they pay taxes. But there is no one to control them and they buy that milk that the large formal collectors do not take because it has antibiotics. The fact is that more control is needed. The dairy farmer learns to use the antibiotic properly if he knows that no one will buy his milk. He does know that if he is caught with antibiotics and takes it to the X or Y cheese factory, they pay him half price, but they pay for it, he does not lose it. So, it doesn't make sense. I have known dairy farmers who send part of their milk to X formal milk collector, and they have a small quota somewhere in a cheese factory where they pour the mastitis milk, that is the withdrawal milk, and the cheese factory receives it, so it is very easy. That's why they don't organize!

Several of the dairy companies in the North of Antioquia also act as suppliers of medicines to dairy farmers. There is no government control over drug suppliers inside and outside the dairy industries. This leads personnel without knowledge in pharmacology, microbiology, or animal health to recommend antibiotics and other medications. This situation promotes the persistence of mastitis, antibiotic resistance in cows, and in bacteria of those who consume milk with antibiotic residues.

V7. There is no government control over the sale of medicines. The drug stores are irresponsible. Who is behind the counter takes the responsibility to give someone a recommendation for treatment without knowing the situation.

#### 3.1.4. Informal Milk Trade

Informal trade is influenced by a series of conditions that lead the dairy farmer to make this decision. Among these conditions is the lack of education and accompaniment for the dairy farmer to build a culture of self-care, and consumer care based on knowledge.

V1. In fact, the last person I would blame for the issue of informal trade is the dairy farmer. First, what we are talking about now is a matter of lack of knowledge. That is, if I am a dairy farmer and I am doing things wrong and someone comes to me and says, “Let's do things this way and clean the udders like this, let's wash the tank, look at the disinfectant,” me, as a dairy farmer, well, that's where my conscience comes in; I say I'm going to do it, and I'm going to do it well. But if I am not instructed and I am not told how to do things, it is difficult. Here there are still very traditional production systems, people who still milk by hand. So, if they don't explain it to me, I'm going to keep doing it the same way my father did it, the same way my grandfather did it.

The high cost of inputs for production conditions the decision-making process regarding the sale of milk in the informal market. Some dairy farmers prefer to sell milk in this market at low cost rather than discard it, in an attempt to cover production costs.

V1. I think that the issue of informal trade can be looked at from many points of view and for example, one that is very important for me because I am involved in it, is, for example, the issue of production costs. Production costs are skyrocketing, very high. Production costs are making people resort to this type of trade.

The lack of tertiary roads makes formal milk marketing difficult; therefore, the primary dairy farmer is forced to process its milk without any sanitary control. These by-products go to the informal and local trade.

V9. Informal trade is favored by difficulties in accessing the farms, lack of tertiary roads, and many farms that are too far away. There are areas where they milk one day and the next day, they take the milk to the tank so as not to go back and forth in the same day. There are areas where they only do a single milking a day, and it does not give them enough time for two milkings. So, for these people, that is their livelihood. Curdling the milk is easier for them than having a tank or selling it to a company that collects the milk cold. I would believe that 50% of the milk goes to cheese right there on the farms, without any control measures, without any drug residue measures, and without any mastitis control measures.

DF5. The peasant must be educated; we must help him with his economy. Peasants in this zone only need a guarantee that they will be able to buy their products and help them with the roads. They do not need anything else because the peasant already survives on the rest.

Problems with the electricity supply in some municipalities promote the informal milk trade. During prolonged power outages, milk spoils, and dairy farmers decide to sell it to collectors who publicly buy withdrawal milk. This is an example of structural problems not dependent on the dairy farmer, whose udder health and milk quality problems require collective action.

V8. Here in Belmira, we have a situation that leads to an increase in informal trade, and that is that the power grid is very fragile. In the last month, in this last winter that began in March, here we could count around 6 or 7 blackouts. There are blackouts that last more than 12 hours. I know dairy farmers who have completely lost the production of two days, those who collect every other day, and the tank tells them: “Man, I'll take it, but they will pay you badly,” or “They will fine you because it is already acidic or because it already has certain problems, it smells bad, look at this or that issue.” So many of the dairy farmers go to certain stores that are actually public because they are not ashamed to say I buy acidic milk, I buy withdrawal milk.

When formal companies begin to demand better milk quality standards from the primary dairy farmer, they can become discouraged, give up their relationship with the company, and take their milk to informal trade milk processors where they do not demand quality parameters. This decision stems from the perception of the dairy farmer's inability to effectively intervene in udder health problems.

DF15. About four years ago, when X company started to squeeze all the dairy farmers for somatic cells, what did many of them do? They went to the cheese factories because they don't get screwed there. In the cheese factory, they are not going to be screwed by somatic cells, by bacteria, and above all by somatic cells, because I can even inject 2 cows that get stung and send the milk. What does the dairy farmer say? X formal company pays me 1200, and in the cheese factory, they pay me 1000, but I don't have to remove the injected cow.

Withdrawal milk containing antibiotics is processed into curd or cheese on the farm and sold on a small scale in the local market by a portion of small dairy farmers.

V2. Some people offer you curds or cheese, which they make on the farm itself. In order not to lose a few pesos, then they simply say no, the withdrawal milk is processed and that is what they give you in the glass of milk.

Another behavior that promotes informal trade is when some collection companies severely punish the dairy farmer for contaminating milk from a tanker truck with milk containing antibiotics. The company charges them the full cost of the contaminated milk. This milk is returned to the dairy farmer; however, fraud networks have been created to guarantee its resale to the informal trade.

V9. There are some companies that punish, let's call it that, but the company does not lose the milk. There are others who tell you, you spoiled 5000 liters, here are these 5000 liters, I am going to pay you the 500 I collected, but you spoiled me 4500 more, you owe me 4500 now, here are your 5000 liters, look for whom to sell them to. So, many times it is already a chain, the driver already has a client to whom to sell it, call it a cheese factory, call it a third party. The same driver of the formal companies talks to the owner of the milk with problems, what does he do? They arrange a price. If the milk was 1200 pesos, they pay around 800 or 700 pesos, the driver earns 100 pesos per liter, and you will not lose so much.

For a dairy farmer, informal collectors represent an alternative to reduce economic loss when they know that their milk will not be bought in the formal trade.

V8. The collector tells you that they will not pick it up because it is acidic milk or it is bad, it has antibiotics for withdrawal. It means losing, for example, 300–400 liters, which is the production of a dairy farmer in two days, because that is a huge hole in your pocket, it means working for free, it means losing money in concentrates and inputs. So, they already have this type of people in their cell phone contacts, who go and pick up the milk and pay it at a low price, but with that at least they get rid of the cost of inputs.

DF8. What do you think about milk being discarded? It is discarded for a reason, for example, 1000 or 1200 liters of milk, to throw that away, imagine! Instead, you call one of those guys, he comes in half an hour, takes the milk, and pays a low price, but well, at least you do not see the milk running through the paddock.

Milk with antibiotic residues that is not discarded on farms is marketed and processed into dairy by-products or local bakery products.

V5. It is very common to have a tank test positive for antibiotics. And there are people who work with that; they work buying milk from dairy farmers who have antibiotics in their tanks and who have tested positive. They buy this milk to process it, to make cheese, or to make their own cheese or bakery products. This is very common, not only here in Entrerríos, but throughout the North, everywhere. Because you cannot lose all the milk, because it is very difficult for a dairy farmer to extract a liter of milk and throw it down the drain, even if it was a mistake to pour milk with antibiotics. What the dairy farmer is looking for is to try not to lose 100% of the milk and to recover some of it.

Informal milk collectors ask about the type of antibiotic in the milk. When the milk contains the antibiotics that they consider the strongest and that can affect cheese production, they do not buy it. There is a concern for the transformation process, but not for the health of the consumer.

DF1. Because instead of throwing the milk on the ground, the cheese factory pays 900 pesos, 800 pesos for it. They come and check what kind of antibiotic it has; that is the first thing they ask because if the one it has is too strong, they don't buy it either. Oxytetracycline is very strong; it does not allow the milk to curdle, which affects the production of cheese.

Informal trade appears to be increasing as it is more profitable for some to process poor-quality milk purchased at low prices, compared to formal market prices. People take advantage of the mastitis problems of the most artisan dairies and have generated a dairy processing company based on informal trade.

DF5. A: There are many people in the informal milk market. In fact, there are people who are starting from scratch, day laborers, as they say from nothing, and at this moment they are already entrepreneurs. They are doing too well buying second-rate milk or milk from farms that do not have cooling tanks or milk with a lot of bacteria, milk with antibiotics or milk with mastitis, and they sell it for 1000 or 1100 pesos.

In addition to the informal milk trade, cows that die or are slaughtered due to chronic mastitis and that have undergone several unsuccessful antibiotic treatments are sold by some dairy farmers to village butcher shops. Said meat quickly reaches human consumption with antibiotic and other drug residues. These actors have established marketing networks that dairy farmers take advantage of to reduce economic losses due to the discarding of cows with complicated mastitis.

V2. You go and look at the prostrate cow; you have treated it with three antibiotics, you have given it everything, it has a lot of withdrawal time, it died, they call the illegal buyer, and that is the same meat that we eat here. If you ask here locally, it is known that there are two or three butcher's shops that are safe; otherwise, all of them use that meat.

Among the actors interviewed in this study, there is a belief that informal trade can exist within formal trade. One way in which this type of trade appears to occur is through the constitution of legal dairy processing companies by large dairy farmers. These production systems, according to the interviewees, take the milk from their cows treated with antibiotics to their own industry to transform it into by-products. The milk from cows that are not undergoing treatment goes to the large formal trade industries.

V2. In a very large farm, the farm produces, for example, 2000 liters, but in those 2000 liters there will always be 5, 10, or 15 cows in withdrawal. They have their own transformation system. So, what do they do? They take out the milk with antibiotics and there it is. What is it? The panequesito that one passes by and buys. So, they don't lose a thousand pesos. In other words, the system is very complete, and they do not see the repercussion beyond that. They looked for an alternative so as not to lose money and obviously, they are big traders, they have the knowledge, and they take advantage of that knowledge. Unfortunately, with serious implications on the health of consumers.

DF8. You see that X formal collection company has Y processing company and there are several companies like that. I worked for 12 years in X collection company, and I know the whole milk process. For example, a tanker car of milk with antibiotics arrives there, that tanker car goes, for example, for industrial cheese or in short, but that is not discarded, that milk is not thrown away.

Professionals who have worked with large dairies state that the milk they receive with antibiotics, with high or low pH, or other contamination, is not discarded and goes into formal commerce. In other words, it constitutes another form of informal trade within the formal trade.

V7. The formal milk collection company receives milk with antibiotics, with whatever residues you want, milk that has a high pH or a very low pH or that has some contamination detected, certain substances to disinfect the tank, peroxide, and then what is happening with that milk? That milk goes to the formal trade.

Dairy farmers affirm that the collection companies that punish them without paying for the milk when the milk contains antibiotics and do not return it dilute it until the antibiotic is undetectable by tests and then use it to make powdered milk for calves.

DF11. They make a mixture with more milk, they take it and test it until they bring it to the level that is needed, undetectable. They pasteurize that milk and make powdered milk for calves.

When formal collection companies suffer from *enlechadas*, where they stop buying all or part of the milk from the primary dairy farmer, the informal collectors take advantage of the situation to buy that milk. This leads dairy farmers to think that the formal companies promote informal trade when they decide not to buy milk from the primary dairy farmer.

DF5. When we are in the time of enlechada, the large companies return the milk to a farmer for whatever reason or stop buying part of it: we are going to receive 200 liters, and the other 100? This is the moment when the farmer who has the means to store milk stores cheese and makes money by selling it later.

One of the events that promote the informal milk trade is the import of milk and whey without use control. This means that the milk collectors do not buy milk from the primary dairy farmer (*enlechada*). This unidirectional commercial interruption by the dairy industry, together with the pressures generated by the dairy farmer's debts, promotes the sale of their milk at a lower price to the informal trade.

DF7. It is necessary to educate the industry and make it more profitable, the government should provide some kind of subsidy for the countryside, for the dairy sector, because times are very hard. When there is an abundance of milk, when there are enlechadas in the country, with imports, the industry subsists. Then, when this type of event occurs on a farm, it is not possible to afford the luxury of losing 100 or 200 liters a day, because in a month there are 3,000 liters. Generally, the whole dairy industry is in debt, so we are forced to sell the milk in one way or another, at a lower price, somehow, but we have to get out of it.

#### 3.1.5. Beliefs about the Elimination of Antibiotics in Milk

There is a mistaken belief among veterinarians that pasteurization eliminates antibiotics in milk.

V1. So, we become very lax, we do not become really rigorous on this issue. We insist a lot: If your milk is not healthy, you cannot send it, despite the fact that the milk collectors carry out pasteurization processes.

Dairy farmers believe that diluting milk with antibiotics in more milk is enough to eliminate the antibiotic or make it undetectable and therefore no longer a health concern for the consumer.

V1. Ah put antibiotic and leave it. If it is contaminated, add milk to the tank to dilute it.

It is mistakenly believed by veterinarians and apparently in the dairy industry that the sum of pasteurization, curdling, heating, and baking of products such as *pandequeso* from milk containing antibiotics can denature the antibiotics.

V3. We do not know how much of the antibiotic can be reported in the cheese bread because it has a pasteurization process, then a curdling process, and then a heating and baking process where I imagine that part or all of the antibiotic has already been denatured.

## 4. Discussion

The findings of the quantitative component indicate some attributes that in their integration and comparison with the theory built in the qualitative analysis allow a better understanding of the phenomena around the formal and informal trade of milk production and the use of milk with antibiotics in udder health and public health frameworks (see Table 3).

### 4.1. Self-Consumption of Milk

The study included dairy farmers who prefer pasteurized milk, considering raw milk a health risk, and others who practice self-consumption of raw milk, unaware of the risks for the consumer.

*Consumption of Raw Milk*. In 2017 figures, milk consumption in Colombian farms corresponded to 7% of the total milk produced, which represents 525 million liters of milk [28]. In different parts of the world, people believe that raw milk has a higher nutritional value than pasteurized milk [17]. Therefore, the self-consumption of raw milk is a practice rooted in the culture of territories [2]. This cultural practice is not only lived in Colombia; consumption of raw milk in the United States is common among peasants, currently varying between 35 and 60%, being preferred for its flavor and nutritional convenience [16]. However, despite the great advances in animal health, milking hygiene, and treatment achieved in recent years, these are not enough to guarantee the microbiological safety of raw milk. There is scientific evidence that milk produced under hygienic conditions sometimes contains significant levels of clinically relevant pathogens [29]. Therefore, the consumption of raw milk is potentially dangerous. The risks and potentially fatal complications, such as neurological and kidney damage associated with raw milk consumption, far outweigh any real or fictitious benefits a consumer might expect to derive from drinking raw milk [29].

*Risk of Zoonosis from Consumption of Raw Milk*. Outbreaks of *Salmonella* spp. associated with the consumption of raw milk have been reported, and despite numerous studies showing the existence of pathogens in raw milk cooling tanks on farms, raw milk continues to be consumed by dairy farming families as a traditional practice [2]. Precisely, some dairy farmers in North Antioquia think that the cooling tank kills bacteria in raw milk, and they did not associate mastitis-causing bacteria with diseases in humans. In this regard, there is sufficient evidence about raw milk as a source of propagation of bacteria such as *Mycobacterium bovis*, *Brucella abortus*, *Staphylococcus aureus*, *Listeria monocytogenes*, *Campylobacter jejuni*, *Salmonella* sp., *Staphylococci* species, and *E. coli,* among others, all of which cause zoonoses [14, 30].

### 4.2. Use of Milk with Antibiotics

Part of the milk with antibiotics that does not go to formal trade can be consumed on the farm by some dairy farming families, although this is not the most common practice.

*Use of Milk with Antibiotics in Calf Feeding*. The use of withdrawal milk in calf feeding is more frequent, based on reducing economic losses due to the withdrawal time of the milk when the cow is being treated with antibiotics. For more than three decades, it has been shown that feeding calves with withdrawal milk containing antibiotics increases resistance to antibiotics in calf intestinal bacteria, especially when the milk contains high concentrations, such as those present at the beginning of a mastitis treatment [31]. This increase in resistance is a concern for both calf health and treatment as well as for humans [31], even more so when bacterial resistance to critically important antibiotics occurs [32]. Resistance to penicillin [31], cefquinome [32], ceftiofur, oxytetracycline, and cephapirin has been described in bacteria from calves consuming withdrawal milk with antibiotics, and multidrug-resistant bacteria have even been isolated [33].

*Discarding Milk with Antibiotics*. Dairy farmers who do not use the withdrawal milk with antibiotic content on the farm, nor send it to the market, discard it on the farm. This action, which in principle has good intentions, can contaminate ecosystems. Environmental contamination with antibiotics in livestock production systems is an important problem for environmental and human health, as it can promote antibiotic resistance in bacteria native to the ecosystem [34]. Exposure to subinhibitory concentrations of antibiotics in environmental matrices has been shown to stimulate a hormone response in bacteria to increase their resilience, which represents a serious threat [34]. Discarding withdrawal milk with antibiotics in natural environments is generally done without any prior intervention to reduce or completely eliminate the antibiotics, which may be affecting soil and water microbiota and, in turn, impacting environmental, animal, and human health [34].

### 4.3. Dairy Industry Control

*Surveillance and Control Mechanism and Actors*. According to resolution 1382 of 2013, the inspection, surveillance, and control of veterinary drug residues in milk is the responsibility of INVIMA [35, 36]. However, the safety of milk from primary production is directly monitored by the dairy industry, which collects milk from each dairy farmer in the formal market and is audited according to the standard by INVIMA [36, 37]. This distribution of power has generated many disagreements and mistrust from dairy farmers towards the dairy industry and the government since the collection companies have the power to be judge and party. Some dairy farmers and veterinarians consider that the companies commit fraud with the laboratory indicators of milk quality, which in turn determine the price of the milk, and especially fraud with the milk they collect with antibiotics and do not return it to the dairy farmer, despite sanctioning it. The study participants recognize INVIMA as an inefficient official control entity in its functions and capacities, monitoring only the largest industries in the formal market and leaving many small and medium-sized companies in formal and informal trade out of surveillance.

*Fraud in the Dairy Chain*. Food safety is a priority issue for any country, and fraud in the food chain is a growing phenomenon today. In a study, 37.18% of Brazilian milk consumers perceived that fraud has always existed, but it was not detected until government surveillance intensified. 26.53% of consumers hypothesized that the cause of fraud would be related to increased competition among the dairy industry, which leads to reduced profits, and fraud becomes an option to increase profits. 80% consider that the industry and transporters are responsible for fraud in the dairy chain [38].

It is curious that scientific interest in fraud has focused on the vulnerability of companies to fraud, without considering the fraud of collecting and processing dairy companies as an option. A study that included dairy farmers, milk transporters, and milk processors suggests that a milk price monitoring system is essential to uncover any abnormal price fluctuations accompanied by advanced fraud monitoring systems and proper detection methods to reduce vulnerability [39]. In addition, food fraud control and prevention can be improved with stricter national food fraud policies [39]. These types of policies are required for the surveillance not only of dairy farmers but also of milk processors. Indeed, the Rural Planning Unit (UPRA) of the Colombian Ministry of Agriculture and Rural Development states that Colombia has a high risk of contamination of products and by-products and that there is insufficient surveillance and control by the responsible entities: INVIMA and health secretariats. In addition, the institutions responsible for supporting the chain in terms of inspection, surveillance and control, training, research, financing, and the operation of the price system are not sufficiently articulated and effective [28].

*Control over Drug Suppliers*. Another result of this study for this category refers to the absence of government control over drug suppliers, who do not comply with the restricted marketing measures with the veterinarian's formula for antibiotics and other drugs such as hormones, anabolics, antimicrobials, analgesics, tranquilizers, anesthetics, muscle relaxants, and other drugs under official control described in ICA Resolution 1167 of 2010 [36]. In addition, untrained personnel recommend antibiotics and drugs without considering some restrictions such as the non-association of bactericidal substances with bacteriostatic substances, the non-acceptance of mixtures of antimicrobials and vitamins, and the prohibition of the use of chloramphenicol as described in ICA Resolution 1326 of 1981 [36].

### 4.4. Informal Milk Trade

*Conditions Favoring Informal Trade and Prevention Strategies*. The informal milk trade in Colombia has not been studied. In this first approach to informal trade from the perspective of those directly involved, the following situations stand out: the high cost of inputs for production, problems regarding access to udder health education, and support for farmers. In addition to the poor tertiary roads that lead to the lack of interest of formal collectors towards the most distant small dairy farms, the difficulties with the electricity supply in some locations and the fact that the lack of surveillance and control in milk imports and the use of whey have consequences on the purchase of the national product. And finally, the option of selling milk with antibiotics to a market without quality requirements and controls. These scenarios impact the sustainability of dairy farmers in their commercial activity and productive tradition. Informal milk trade has already been described as the livelihood of dairy farmers in Kenya, a country with about 86% informal trade [40]. For the Colombian case and based on the reality of North of Antioquia, these situations have not been considered with specific and forceful strategies in dairy sector policies. There is sufficient scientific evidence to support the need for integrated policies on udder health, informal trade, and public health in Colombia. Indeed, in Kenya, synergistic efforts between research and programs with dairy farmers have contributed to the implementation of policies and their evaluation [40]. Processes such as knowledge transfer of programs and policies, theoretical and practical training, education in milk quality control, collective action among actors, and the risks of informal trade for public health have been described as fundamental in this type of sociocultural transformation [40].

The frauds described by dairy farmers from the 9 municipalities participating in the study, such as the addition of whey to milk by the dairy industry to reduce milk collection in the North of Antioquia, are some of the reasons given that have encouraged the sale of milk from primary production to the informal market. In accordance with this scenario, in the Brazilian dairy chain, fraud due to the addition of substances to increase the quantity of milk and to reuse deteriorated milk or prolong its shelf life has been shown to have an impact on the reduction of milk consumption, damage to the image of the dairy sector, and promotion of the informal milk trade [38].

In other areas of Colombia, the reasons that promote informal trade are not different from those described in the North of Antioquia: the entry of dairy products into the country through the free trade agreement (FTA) with the United States; the size of the production systems, which is directly related to financial resources and their ability to invest and reduce costs; and the poor condition of secondary and tertiary roads decreases the interest and commitment of formal processors to collect milk from remote farms [41]. In their intention to remain in business, these conditions influence dairy farmers, promoting the production of cheese on the farm for small-scale trade and the sale of raw milk to cheese makers in the area or as liquid milk to local peasant families, which is well received by the communities due to their consumption habits and a lower cost per liter of raw milk compared to pasteurized milk [41].

*Planning in Udder Health as a Strategy to Prevent Informal Trade*. To a large extent, milk safety problems are related to its purchase in the informal market and previous events. Some of the elements considered in programs to improve milk safety and quality in some countries include intervening in problems that promote informal trade with strategies such as training dairy farmers in hygienic production; organizing dairy farmers into dairy cooperatives; linking service centers to improve milk production; supporting the processing and retailing of formal trade; training and legitimization of actors in the informal sector; raising consumer awareness of milk safety; implementation of technologies to diagnose and treat mastitis appropriately; access to milk preservation technologies; access to information technology and implementation of strategies to enforce milk safety legislation; development of infrastructure to improve roads, water, and electricity; and promotion of peasant education from primary and secondary education [42].

### 4.5. Beliefs about the Elimination of Antibiotics in Milk

*Heat Treatments of Milk for Human Consumption*. Raw milk is generally subjected to heat treatment prior to processing and consumption to reduce the amount of zoonotic pathogenic bacteria and increase the shelf life of the product. The dairy industry uses thermization, pasteurization, ultra-high temperature treatment, and sterilization as heat treatments of raw milk. In household conditions, raw milk is generally treated by boiling. All these treatments are time and temperature dependent [43].

*Degradation Rates of Antibiotics in Milk Subjected to Heat Treatments*. For the consumption of milk on farms in the North of Antioquia or by consumers in rural areas, people usually heat treat milk by boiling it for a few minutes as a hygienic method. This practice is inefficient when the milk contains antibiotics since the thermal stability between antibiotics is variable. An experimental study that evaluated the degradation of residues of 11 antibiotics in milk obtained the following degradation percentages when treating the milk at 100°C for 5 minutes: ampicillin (26.8%), cloxacillin (9.6%), penicillin-G (43%), cefoperazone (78.3%), cephalexin (37.4), streptomycin (43.8%), neomycin (43 0.6%), tetracycline (30.4%), trimethoprim (22.6%), sulfadiazine (27.6%), and sulfathiazole (30.1%). These results demonstrate that antibiotic residues in raw milk have high heat stability when treated for about 5 minutes at 100°C [43]. Treatment for a few minutes as a protective measure for the consumption of withdrawal milk with antibiotics is not enough because it does not completely eliminate antibiotics, putting the consumer's health at risk [43].

*History of Antibiotic Residues in Raw and Pasteurized Milk*. Data compiled by the Colombian Ministry of Health and Social Protection-INS for 2011 found that between 1990 and 2010, 19 studies in Colombia evaluated the presence of antimicrobials in milk. Most of these studies focused on the identification of beta-lactams, tetracyclines, and sulfonamides. The prevalence of antimicrobial residues in milk varies according to the study [44]. For 2011, they reported results from 3 studies. In Cordoba, they evaluated 445 samples of raw milk and pasteurized milk, finding 25% positive samples. In Sucre, out of 2110 raw milk samples, 7.73% of the samples were positive for antimicrobials. In Bogotá, out of 165 samples, 23.64% of the milk analyzed presented beta-lactam residues [44]. In other countries, the findings are worrisome. In Kenya, antibiotics were found in 8% of raw milk and 8.2% in pasteurized milk. In Mexico, the results for sulfonamides in four pasteurizing companies show figures of 47.2%, 58.3%, 44.7%, and 50%, respectively. In Peru, 20.67% of positive samples for antibiotics were found in milk from markets and 21.21% from stores and farms. In Italy, 49% of the samples were positive for penicillin-G, 5.6% for amoxicillin, and 3.8% for cephalosporins. Finally, in Brazil, a large milk processor found 30.8% of milk samples with antibiotic residues, of which tetracyclines, neomycin, beta-lactams, gentamicin, chloramphenicol, streptomycin, and dihydrostreptomycine were the antibiotics found [44].

*Effects of Antibiotics and Their Metabolites in Drinking Milk*. Reducing the use of antibiotics and education on a rational use in animal production is fundamental. It has been found that not only the presence of antibiotics in milk, but also their metabolites and/or processing products produced in the heat treatments of sterilization (120°C/20 min) and pasteurization (60°C/30 min or 72°C/15 s), may be responsible for bacterial resistance, allergy, and/or toxicity in humans [45]. For example, residues of compounds containing *β*-lactam rings can lead to bacterial resistance, and some degradation products, such as penicilloic acid, induce allergy and toxicity in sensitized individuals [46].

*Withdrawal Milk and Cheese Production*. In Colombia, half of the milk with antibiotics that goes to the informal trade is processed into cheese [28]. This activity is reported by dairy farmers and veterinarians in the North of Antioquia as a traditional activity in an attempt to reduce economic losses due to milk withdrawal times [47]. There is a belief that antibiotics are broken down during the cheese-making process. In turn, some scientific advances oppose this belief. A study in Brazil analyzed the degradation of monensin residues in the cheese-making process. No significant degradation of monesin was observed due to heat treatment, suggesting that the antibiotic is thermostable at high temperatures. Furthermore, the residue levels quantified in cheese and whey showed a concentration of this antibiotic in the curd of approximately 5 times, i.e., the antibiotic was concentrated. Additionally, physicochemical parameters and cheese fermentation were not affected by the presence of the antibiotic [48]. These studies have provided sufficient evidence demonstrating that heat treatments performed on milk, such as pasteurization or sterilization, do not completely eliminate antimicrobial residues. Therefore, they are insufficient as a control measure to prevent them from reaching the consumer [49]. The use of antibiotics in cattle represents a risk to public health and raises the need for strategies for udder health planning to reduce the use of antibiotics.

## 5. Conclusions

There are several situations that exert pressure on dairy farmers, compelling them to make decisions regarding the informal trade of milk with antimicrobial residues. Some of these situations include economic difficulties of primary production and low profitability, limited education, lack of tertiary roads, problems with electricity supply in some areas, poor milk quality and the lack of demand for it by informal collectors, promotion of this trade by informal and formal collectors, milk imports and inappropriate use of whey by the industry, the absence of state control over the dairy industry, and suppliers of animal drugs. At the same time, the self-consumption of withdrawal milk and the use of milk with antibiotics in calf feeding is part of the culture of dairy farmers and their families. In contrast, only some of them discard milk from cows treated with antimicrobials, which is insufficient, since they fail the protocols, contaminating soil and water. These attitudes that define trade and consumption actions occur in the context of ignorance, given that heat treatments of milk are inefficient in the complete elimination of antibiotics. This complex problem promotes bacterial resistance in animal and human bacteria, as well as in soil and water bacteria, posing a subsequent risk of consuming water and vegetables with antimicrobial residues. This represents, from a single health analysis, an impact on the three links: environmental, animal, and human health. The findings of this study show that the effects on udder health constitute a public health problem that not only involves the dairy farmer but also other actors in the dairy chain that promote a culture of irrational use of antibiotics, inappropriate use of milk, and informal trade. A cultural transformation is required in dairy farmers, veterinarians, the dairy industry, drug suppliers, informal traders, and the consumer. These cultural transformations should be encouraged by public policies, which, through programs and strategies, aim to provide control while simultaneously educating on good production practices, udder health, commercialization, processing, regulated use of antibiotics, and milk consumption.

## Figures and Tables

**Table 1:** Bulk milk quality according to bulk tank milk somatic cell count (BTSCC) and colony-forming units (CFUs).

	BTSCC			CFU	
	*n*	%		*n*	%

Excellent. Less than 150,000	3	1.4	Excellent. Less than 75,000	117	54
Good. 150,001 to 250,000	19	8.8	Good. 75,001 to 150,000	39	18
Acceptable. 250, 001 to 400,000	48	22.2	Acceptable. 150,001 to 250,000	12	6
Deficient. Greater than 400,000	146	67.6	Deficient. Greater than 250,000	48	22

**Table 2:** Antibiotic use, marketing, antibiotics in milk, and services for mastitis control and prevention.

		%	BTSCC Median (IQR)	CFU Median (IQR)
When I am administering antibiotics to the cow with mastitis, I consider that it is okay to consume its milk at home or sell it in the village	Disagree	98.6	495156 (367558–670480)	66400 (22682–206622)
	Agree	1.4	436377 (375678–767625)	25706 (10444–53758)

I am concerned about consumer health if I sell milk from cows in the village that the dairy companies would not buy from me	Disagree	0.5	654786 (654786–654786)	9000 (9000–9000)
	Agree	99.5	495089 (367558–670489)	63622 (22682–206622)

Reported type of marketing of the milk it produces	Formal trade	98.6	490882 (367558–670480)	56797 (22682–169444)^*∗*^
	Informal trade	1.4	670480 (670480–670480)	303134 (303134–303134)^*∗*^

Intention to sell milk in the village when the dairy industry does not buy it because of high BTSCC	Disagree	74.1	542100 (387169–685897)^*∗*^	69856 (23971–216341)
	Agree	25.9	457056 (298846–639611)^*∗*^	39561 (21774–112738)

^*∗*^ *p* < 0.05.

**Table 3:** Matrix for comparison of similarities and integration of qualitative and quantitative results.

Variable	Attributes	Theory (based on qualitative analysis)
When I am administering antibiotics to the cow with mastitis, I consider that it is okay to consume its milk at home or to sell it in the village	98.6% disagree BTSCC and CFU higher for those who disagree	*Self-consumption of milk* Milk produced on the farm is an essential food for families Some dairy farmers prefer pasteurized milk because of the risks of raw milk Many are unaware that bovine mastitis bacteria can affect human health Some believe that the cooling tank kills bacteria in raw milk *Use of milk with antibiotics* In calf feeding Self-consumption on the farm Those who put their ethics first discard milk. However, protocols are required to avoid soil and water contamination.

Intention to sell milk in the village when the dairy industry does not buy it for high BTSCC	25.9% agree to sell it despite poor quality BTSCC and CFU higher for those who disagree on selling milk with poor sanitary quality in the village	*Control of the dairy industry* Government control over the quality of milk is only applied to large dairy and processing industries There is no government control over drug suppliers

Type of informed selling of the milk produced	1.4% report in surveys that they trade informally CFU and BTSCC higher for those who do informal trade	*Informal milk trade* In interviews, most dairy farmers reported informal trade of milk containing antibiotics Dairy farmers' decisions about milk marketing are influenced by the following factors: Lack of education and accompaniment to the dairy farmer High cost of inputs The lack of tertiary roads makes formal milk marketing difficult Problems with electricity supply Some dairy farmers prefer to sell their milk to a company that does not demand quality milk Milk with antibiotics is marketed in liquid form, or transformed into cheese, curds, or bakery products Tank truck drivers who return milk with antibiotics to the dairy farmer promote informal trade Informal collectors represent an alternative to reduce the economic loss when they must discard milk Informal trade is increasing as it is more profitable to process poor-quality milk bought at low prices Meat from cows with chronic mastitis subjected to antibiotics is taken to the informal trade Large dairy farmers generate processing companies where they take their withdrawal milk Informal trade within the formal trade by medium and large milk collection and processing companies Imports of milk and whey promote *enlechadas* and the informal trade

I am concerned about consumer health if I sell milk in the village from cows that dairy companies would not buy from me	99.5% are concerned Higher BTSCC and lower CFU in milk from farms whose dairy farmer is not concerned about consumer health	*Beliefs about the elimination of antibiotics in milk* The mistaken belief that pasteurization eliminates antibiotics in milk It is believed that diluting milk with antibiotics in more milk is sufficient and does not impact health It is believed that the addition of pasteurization, curdling, heating, and baking of products such as *pandequeso* denatures antibiotics completely

## Data Availability

All data pertaining to the current study are available from the corresponding author upon reasonable request.
